# Editorial: Physical activity as a natural cure for non-communicable diseases

**DOI:** 10.3389/fpubh.2023.1209569

**Published:** 2023-05-31

**Authors:** Bojan Masanovic, Selcuk Akpinar, Szabolcs Halasi, Dušan Stupar, Stevo Popovic

**Affiliations:** ^1^Faculty for Sport and Physical Education, University of Montenegro, Niksic, Montenegro; ^2^Western Balkan Sport Innovation Lab, Podgorica, Montenegro; ^3^Faculty of Sports Science, Nevşehir Haci Bektaş Veli University, Nevşehir, Türkiye; ^4^Hungarian Language Teacher Training Faculty, University of Novi Sad, Subotica, Serbia; ^5^Faculty of Sport and Psychology, Educons University, TIMS, Novi Sad, Serbia

**Keywords:** exercise, healthy lifestyle, children, adults, older adult, chronic diseases

## 1. Introduction

Despite the numerous epidemiological studies demonstrating the health benefits of physical activity and the increased risk of chronic diseases associated with physical inactivity, a significant portion of the global population remains physically inactive (1–4). The modern era of industrialization and communication has resulted in a lifestyle shift and reduced physical activity across all age groups (5, 6). Physical activity and physical fitness are recognized determinants of health, and thus the negative health consequences resulting from physical inactivity are not surprising (7). The global burden of non-communicable diseases (NCDs), responsible for over 40 million deaths annually (8), is alarming, particularly in individuals aged 30–69 years (15 million deaths per year). This highlights the need to focus on the significant social issue of physical inactivity as part of the United Nations 2030 agenda. Given the negative trends of modern lifestyles, it is essential to develop strategies to promote physical activity behavior and understand the genetic, physiological, environmental, and behavioral factors related to major NCDs. Consequently, the objective of this Research Topic is to further develop knowledge on the effects of physical (in)activity on major NCDs and improve our understanding of the factors involved. In conclusion, physical inactivity is a significant public health issue that requires urgent attention. The development of effective strategies to promote physical activity and reduce the incidence of NCDs is essential. A deeper understanding of the underlying mechanisms and risk factors involved in NCDs will enable the identification of new therapeutic and preventative approaches and contribute to the development of effective public health strategies.

## 2. Contribution to the field

The aim of this Research Topic is to gather and disseminate new knowledge pertaining to the impact of physical (in) activity on major non-communicable diseases across all age groups, ranging from childhood to old age. The 10 studies that have emerged as a result of this Research Topic, comprising six cross-sectional studies, one case-control study, and three review articles, offer readers a unique opportunity to expand their knowledge in this field. The findings presented in these studies are expected to advance our understanding of the relationship between physical activity and non-communicable diseases, thus contributing to the development of effective strategies to prevent and manage these diseases. By focusing on a range of age groups, these studies provide a comprehensive view of the impact of physical activity on health outcomes across the lifespan, offering valuable insights into the potential benefits of physical activity promotion programs for individuals of all ages.

The majority of the studies conducted in this Research Topic primarily focused on adults. However, to establish a comprehensive understanding of the impact of physical (in)activity on non-communicable diseases, it is crucial to also investigate its effects on the youngest population, i.e., children. This Research Topic included three studies that examined the effects of physical activity on children aged 5–17 years. These studies, consisting of two cross-sectional studies and one case-control study, evaluated the benefits of physical activity on gross motor skills, physical fitness, sensory integration, kinetic visual acuity, uncorrected distance visual acuity, and strength performance. The first study by Fu et al. investigated the effects of functional training, lasting 12 weeks, on children's gross motor skills, physical fitness, and sensory integration. The results of this study showed a significant improvement in all three areas, highlighting the benefits of functional training for children's physical development. The second study by Yin et al. examined the effects of physical activity combined with extra ciliary-muscle training, lasting 16 weeks, on children's kinetic visual acuity and uncorrected distance visual acuity. The results showed that the combination of physical activity and extra ciliary-muscle training improved both visual acuity measures, indicating the potential benefits of this intervention for children's vision. The third study by Patti et al. assessed the effects of physical exercise continuity during the COVID-19 pandemic on strength performance in children. The results indicated that consistent physical exercise during the pandemic period resulted in higher strength performance in both the Handgrip test and the Countermovement Jump test. Overall, these studies provide important insights into the benefits of physical activity for children's physical development, particularly in the areas of gross motor skills, physical fitness, sensory integration, vision, and strength performance.

The following five studies investigated the effect of physical activity on a sample of adults aged 18–60 years. The first three studies, which were cross-sectional in design, examined the impact of physical activity on various health outcomes, all concluding that physical activity has a positive effect. One study found that physical activity could reduce the incidence of kidney stones in diabetes patients with a high body mass index (BMI), despite high BMI being a risk factor for kidney stones (Mao et al.). Another study reported that greater participation in mass sports increased the likelihood of prosocial behavior (Duan et al.), while a third study found that increased physical activity could prevent hypertension (Zhou et al.). The remaining two studies, which were review articles, aimed to determine the potential of physical activity in suppressing the negative effects of sedentary behavior on cardiovascular disease and obesity epidemics (Liang et al.; Rizzato et al.). The results of these studies suggest that physical activity can reduce the risk of cardiovascular disease and improve indicators associated with it. Furthermore, alternative workstations, such as standing or walking workstations, seated pedals, and gymnastic balls, may be useful in combating the obesity epidemic.

An additional study in this Research Topic focused on both adults and older adult individuals. It was a systematic review study that examined a clinical population with participants ranging from 48 to 75 years of age. The study concluded that physical exercise interventions may improve, or at least not worsen, cognitive performance in patients undergoing hemodialysis (Bogataj et al.).

The last study was a cross-sectional study that examined individuals in the older adult age group (65–85 years). The study aimed to investigate the combined impact of smoking and physical activity on mortality rates in older adult patients diagnosed with hypertension. The study's results suggest that the combination of smoking and physical inactivity may have a synergistic effect on the risk of premature death, emphasizing the critical need to enhance behavioral factors and advocate for a comprehensive healthy lifestyle in older adult patients with hypertension (Yang et al.).

## 3. Conclusion

This particular Research Topic contains 10 articles that support well-established evidence regarding the positive effects of regular physical activity on health outcomes (9). In addition, the Research Topic provides specific recommendations in the form of prepared physical exercise programs, which can be found in the published articles. These programs have undergone verification and can be implemented, either in full or in part, by practitioners to prevent, treat, or alleviate certain health conditions. Moreover, there is a high likelihood that these programs will lead to a predicted transformation or output state. It is highly probable that the information and practical suggestions presented in this Research Topic will inspire researchers to develop even better solutions to combat Non-communicable Diseases.

## Author contributions

BM and SA drafted the Editorial. SP, SH, and DS revised and approved the final version. All authors contributed to the article and approved the submitted version.
